# Diagnosing Intraoperative Pneumothorax in Patients Undergoing Autologous Breast Reconstruction: A Useful Clinical Sign

**DOI:** 10.1155/2014/308485

**Published:** 2014-08-12

**Authors:** Thomas Reekie, David McGill, Elizabeth Marshall

**Affiliations:** ^1^Canniesburn Plastic Surgery Unit, Glasgow Royal Infirmary, Glasgow, UK; ^2^Glasgow Royal Infirmary, Glasgow, UK

## Abstract

Intraoperative pneumothorax during breast reconstruction can be difficult to diagnose. Even a small pneumothorax can become a tension pneumothorax under positive pressure ventilation. The clinical finding of venous congestion in a pedicled latissimus dorsi flap, which could not be explained by problems with the vessels, preceded other signs of a tension pneumothorax in the case presented here. Given the difficulties of access to the chest by the anaesthetic team during breast procedures, this has the potential to be a useful adjunct in the diagnosis of this potentially serious intraoperative complication.

## 1. Introduction

Pneumothorax is a potentially dangerous intraoperative complication, which can be difficult to diagnose [1]. Here we present a case of an intraoperative pneumothorax during breast reconstruction which demonstrated a useful clinical sign to aid diagnosis.

A 67-year-old patient attended clinic to be considered for delayed breast reconstruction following left mastectomy two years previously for widespread ductal carcinoma in situ. Sentinel lymph node biopsy following completion mastectomy showed 0/6 nodes positive. The patient had not required chemotherapy or radiotherapy. Medical comorbidities included well-controlled type 2 diabetes mellitus and hypertension. She had a good exercise tolerance, with no history of COPD or asthma, and had previously undergone appendicectomy, salpingooophorectomy, and hysterectomy with no anaesthetic complications. The patient is a lifelong nonsmoker.

Chest X-ray was performed to investigate left sided chest pain 12 weeks pre-op and was noted to be grossly normal. After discussion with the senior surgical author in clinic, the patient opted to undergo an extended latissimus dorsi breast reconstruction with a contralateral symmetrising mastopexy.

The patient was admitted the day before the procedure and was seen by the anaesthetic team the evening before the operation. No concerns were noted.

In theatre, the patient was given a general anaesthetic and was intubated with a cuffed endotracheal tube and pressure control ventilation commenced. No central venous access was attempted at any stage.

**Figure 1:**
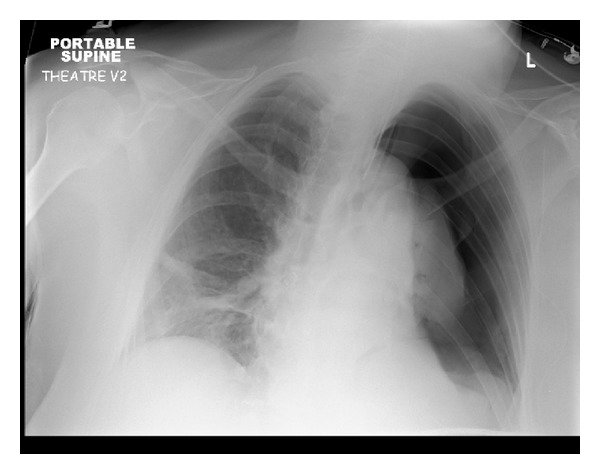
Chest X-ray taken ten minutes after partial decompression of left sided tension pneumothorax (patient still requiring positive pressure ventilation).

A left extended latissimus dorsi musculocutaneous flap was raised in the standard fashion and transposed to reconstruct the left breast. The pedicle was checked to ensure it was not twisted; the flap was inset and appeared healthy. Towards the end of the procedure, the flap started to show signs of venous congestion. During this time, the anaesthetist noted slowly progressive hypotension, tachycardia, and low pulse oximeter readings associated with increased ventilation pressures. The patient was recovering at this point and was coughing, thus requiring increased ventilation pressures and providing an erroneous explanation for the drop in oxygen saturations. Despite difficulties with auscultation owing to the surgical site, reduced air entry was noted on the left side. A left sided tension pneumothorax was diagnosed and needle aspiration of the pleural space performed, with initially good physiological response, but no audible decompression. Whilst setting up the chest drain, a chest X-ray was performed (see Figure 1) and a second needle thoracostomy performed, resulting in an audible hiss and physiological improvement. The chest drain was placed and the patient remained stable.

After this intervention, the venous congestion seen in the flap rapidly resolved. The patient made a good recovery and had the chest drain removed three days postoperatively. She was discharged when fully recovered seven days later.

## 2. Discussion

Even a small pneumothorax can become a tension pneumothorax when positive pressure ventilation of the lungs is delivered during general anaesthesia [1]. Pneumothorax is a rare complication during breast surgery, with an incidence of less than 1% [2]. A higher incidence is reported during secondary breast surgery or breast reconstruction but still remains a relatively rare event [2]. To try and further limit the likelihood of pneumothorax during these procedures, care should be taken when raising the flap and if quilting the latissimus dorsi donor site, or staples can be used to mark out the breast footplate instead of a needle to reduce the risk of inadvertently entering the pleural cavity. Despite being aware of these measures in this unit, a recent audit of pneumothorax following extended latissimus dorsi flap breast reconstruction found an incidence of 0.4% [3]. Bacon et al. note the difficulty of diagnosing pneumothorax intraoperatively and advocate the use of a specific pneumothorax algorithm to enable expedient diagnosis and management, highlighting the rarity of the condition as a need for a high index of suspicion and systematic approach [1]. Many of the parameters that are recommended in this checklist are hampered by the surgical site, as the whole chest is normally prepped to allow for comparison of breast symmetry. Palpation, percussion, and auscultation of the chest or use of the finger method described by Tsarev et al. [4] are not possible without entering the sterile surgical field. The surgical drapes also cover the abdomen and majority of the trachea, further hindering examination by the anaesthetic team.

In light of this, any additional information which can be provided by the operating surgeon would be beneficial for the diagnosis. Here, the congested flap seen intraoperatively was an indicator of the underlying pathology. As the patient was coughing and ventilation pressures became higher, the pneumothorax likely then became a tension pneumothorax with the attendant marked hypoxia and tachycardia.

The latissimus dorsi flap used in delayed breast reconstruction often includes a paddle of skin and subcutaneous fat which is used to reconstruct the breast skin envelope, and this also provides a means of monitoring the flap. The vascular pedicle for this musculocutaneous flap is the thoracodorsal vessels, which are one of two branches from the subscapular vessels (the largest branch of the axillary artery and vein). A sustained increase in intrathoracic pressure, as seen in a pneumothorax, could result in impaired venous return from these vessels and thus venous congestion of the flap. Once torsion of the pedicle has been ruled out, a potential intrathoracic pathology should be considered. Given that these flap changes were evident before changes in blood pressure and oxygen saturation, which are in themselves nonspecific [1], new unexplained intraoperative flap venous congestion could be considered a useful early sign for diagnosing a pneumothorax. Indeed, in an animal study of pneumothorax by Rutherford et al., an increase in the right side of the circulation pressure with maintained cardiac output was seen during an induced pneumothorax long before it became a tension pneumothorax [5], thus supporting the notion that flap venous congestion could be seen before dramatic changes in the patient's observations.

During free tissue transfer for breast reconstruction, the internal mammary vessels are often used as recipient vessels for the flap. Pneumothorax is more of a risk given the close proximity to the pleura, prompting a greater awareness of the potential diagnosis. However, if a spontaneous pneumothorax were to occur, then flap congestion could potentially be seen due to increased intrathoracic pressure impeding venous return in a similar manner to that already described.

## 3. Conclusion

Expediently diagnosing a pneumothorax remains a challenge to all staff involved in the patient's intraoperative care. Although algorithms exist to aid in the diagnosis, patients undergoing breast reconstruction may prove difficult to assess due to limited access and initially nonspecific signs. Here, we present an unusual surgical finding which appears to be a useful addition for expediently diagnosing a serious complication in a challenging patient group.
